# Discovery and validation of the tumor-suppressive function of long noncoding RNA PANDA in human diffuse large B-cell lymphoma through the inactivation of MAPK/ERK signaling pathway

**DOI:** 10.18632/oncotarget.20053

**Published:** 2017-08-07

**Authors:** Yingjun Wang, Mingzhi Zhang, Huanan Xu, Yifei Wang, Zhaoming Li, Yu Chang, Xinhuan Wang, Xiaorui Fu, Zhiyuan Zhou, Siyuan Yang, Bei Wang, Yufeng Shang

**Affiliations:** ^1^ Department of Oncology, The First Affiliated Hospital of Zhengzhou University, Lymphoma Diagnosis and Treatment Center of Henan Province, Zhengzhou, Henan, China; ^2^ Department of Anorectal Surgery, The First Affiliated Hospital of Zhengzhou University, Zhengzhou, Henan, China; ^3^ Department of Ultrasonography, The First Affiliated Hospital of Zhengzhou University, Zhengzhou, Henan, China

**Keywords:** lncRNAs, diffuse large B-cell lymphoma, lncRNA PANDA, p53, MAPK/ERK

## Abstract

Diffuse large B-cell lymphoma (DLBCL) is one of the leading causes of cancer-related mortality, and responds badly to existing treatment. Thus, it is of urgent need to identify novel prognostic markers and therapeutic targets of DLBCL. Recent studies have shown that long non-coding RNAs (lncRNAs) play an important role in the development of cancer. By using the next generation HiSeq sequencing assay, we determined lncRNAs exhibiting differential expression between DLBCL patients and healthy controls. Then, RT-qPCR was performed for identification in clinical samples and cell materials, and lncRNA PANDA was verified to be down-regulated in DLBCL patients and have considerable diagnostic potential. In addition, decreased serum PANDA level was correlated to poorer clinical outcome and lower overall survival in DLBCL patients. Subsequently, we determined the experimental role of lncRNA PANDA in DLBCL progression. Luciferase reporter assay and chromatin immunoprecipitation assay suggested that lncRNA PANDA was induced by p53 and p53 interacts with the promoter region of PANDA. Cell functional assay further indicated that PANDA functioned as a tumor suppressor gene through the suppression of cell growth by a G0/G1 cell cycle arrest in DLBCL. More importantly, Cignal Signal Transduction Reporter Array and western blot assay showed that lncRNA PANDA inactivated the MAPK/ERK signaling pathway. In conclusion, our integrated approach demonstrates that PANDA in DLBCL confers a tumor suppressive function through inhibiting cell proliferation and silencing MAPK/ERK signaling pathway. Thus, PANDA may be a promising therapeutic target for patients with DLBCL.

## INTRODUCTION

Diffuse large B-cell lymphoma (DLBCL) occurs most commonly in all subtypes of non-Hodgkin lymphoma (NHL), representing more than one-third of all diagnosed NHL cases and making it the most prevalent form of NHL among adults worldwide [1, 2]. The administering of the regimen including rituximab plus cyclophosphamide/doxorubicin/vincristine/prednisone (R-CHOP) has been seemed as the standard therapy for the patients with DLBCL, remarkably improves the prognosis [3, 4]. However, a great amounts of patients still suffer from unsatisfactory prognosis [5]. Thus, it is important to investigate novel biomarkers involved in DLBCL development.

Gene expression profiling of specimens from DLBCL has revealed broad gene expression deregulation compared to healthy individuals. It is more and more clear that most of the genome DNA is represented in processed transcripts without or lacking of protein-coding capacity [6]. Long noncoding RNAs (lncRNAs) have been implicated in a variety of physiological and pathological processes. In cancer, aberrant expression and mutations of lncRNAs can contribute to tumor development and progression by promoting proliferation, invasion, metastasis, and survival [7, 8]. Several groups have performed systematic analysis of lncRNAs by Hiseq sequencing array, a high throughput screening method, in normal cells and also in primary tumors. A recent cross-cancer study by the Chinnaiyan group uncovered thousands of novel lncRNAs [9]. Another study by the Maher group identified a large number of novel lncRNAs in lung cancer [10].

Dysregulation of some lncRNAs is well accepted as prognostic factor and therapeutic target for specific cancers. For example, HOTAIR contributes to the tumorigenesis of breast cancer [11], MALAT1 is attributed to the metastasis of non-small cell lung cancer [12], and HULC is identified as critical regulator of pancreatic cancer [13]. The long non-coding RNA PANDA (P21 Associated ncRNA DNA Damage Activated) is located ∼5 kb upstream of the CDKN1A (p21) transcription start site, is evolutionarily conserved, specifically induced by DNA damage and mediates proliferation/apoptosis functions [14]. It is reported that PANDA positively regulates proliferation of osteosarcoma cells [15]. However, the patterns and biological function of lncRNA PADNA in DLBCL is still unclear.

In this study, a high-throughput Hiseq sequencing was firstly performed to find the potential aberrant lncRNAs. Then, the reverse transcription quantitative real-time PCR (RT-qPCR) assays were used to validate the upregulation or downregulation of those lncRNAs in both clinical samples and cell materials. We reveals that lncRNA PANDA is downregulated in DLBCL patients and the suppression of PANDA indicates poor outcome. Moreover, lncRNA PANDA can inhibits proliferation of DLBCL cells through the inactivation of MAPK/ERK signaling pathway.

## RESULTS

### Discovery of potential lncRNAs by Hiseq sequencing method

**Figure 1:**
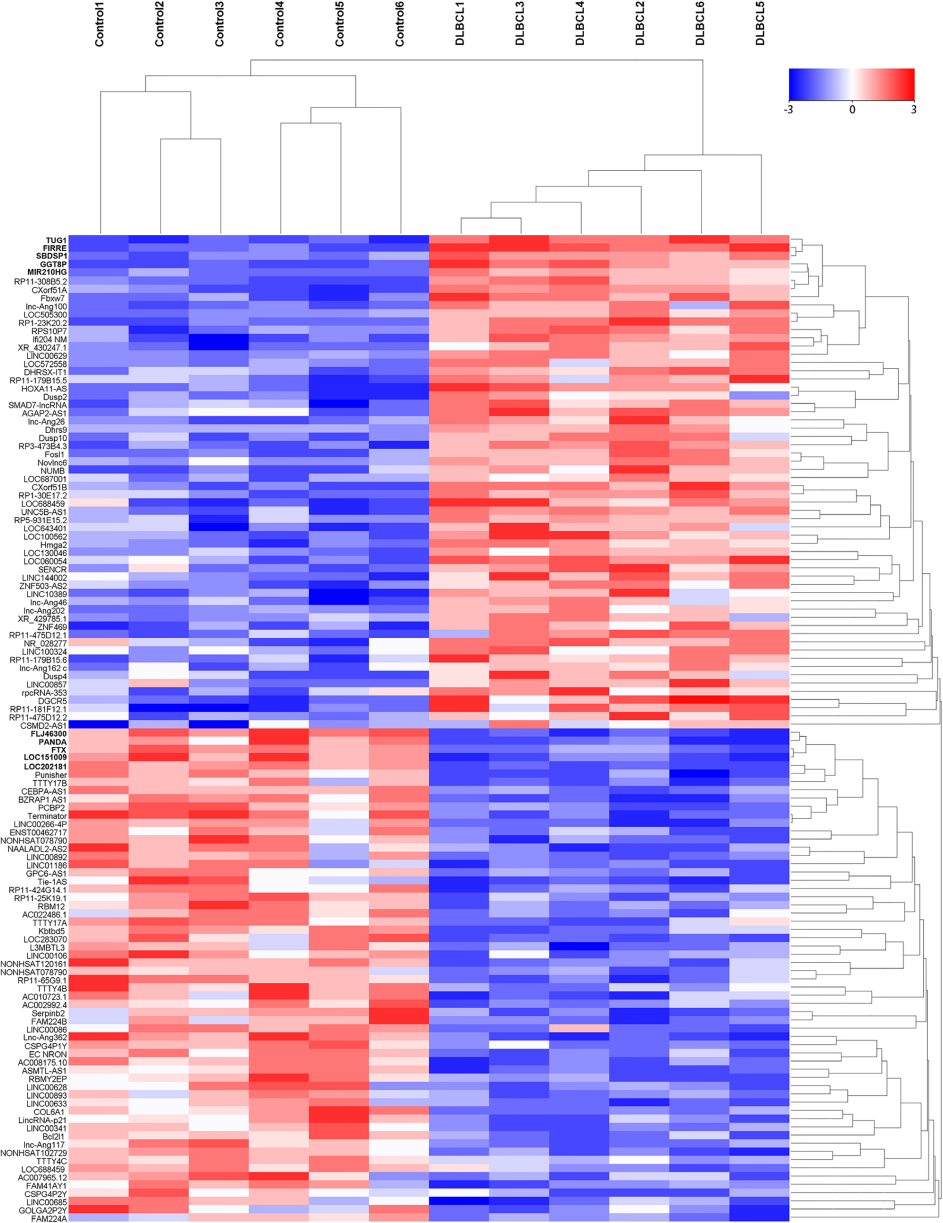
The heat map shows expression of the 120 lncRNAs most up- or down-regulated in DLBCL patients compared with healthy controls The top 60 lncRNAs up- and down-regulated in non-responding group are shown in the top and bottom halves, respectively. The heat map was generated with an R package using normalization across rows (serum samples).

We firstly screened potential lncRNAs that differentially expressed between DLBCL patients and healthy individuals via Hiseq array in the discovery phase. On the basis of the date obtained from Hiseq sequencing, we then identified 546 lncRNAs that were differently expressed more than 2-fold change. Subsequently, we narrowed the scope of the study to the 120 most aberrant expressed RNAs, including 60 up-regulated lncRNAs and 60 down-regulated lncRNAs (Figure 1). These lncRNAs should be easily detected with a Ct value less than 35, easily designed primers and have stable experssion in both primary tissues and serum samples. According those definition, we finally restricted to 10 lncRNAs from 5 of up-regulated group and 5 of down-regulated group (Table 1). In addition, another five lncRNAs were brought into our study, as they were reported to have potential function during DLBCL progression [16–20]. Thus, we discovered 15 candidate lncRNAs which may be potential biomarkers for DLBCL patients, pending further validation.

**Table 1:** Candidate lncRNAs selected on a basis of the Hiseq analysis

Seq-name	Location	Regulation (D. vs C.)	Fold change	*P* value
TUG1	Chr22q12.2	Up	112.1462	0.00007984
FIRRE	ChrXq26.2	Up	77.3592	0.00016824
SBDSP1	Chr7q11.23	Up	39.0431	0.00082395
GGT8P	Chr2p11.2	Up	31.0532	0.00090357
MIR210HG	Chr11p15.5	Up	25.3981	0.00163746
FLJ46300	Chr10q26.3	Down	65.4768	0.00015396
PANDA	Chr6p21.1	Down	43.6921	0.00044281
FTX	ChrXq13.2	Down	29.4650	0.00103498
LOC151009	Chr2q13	Down	17.3986	0.00204437
LOC202181	Chr5q35.3	Down	14.6935	0.00411932

D.: DLBCL; C.:Controls.

### LncRNA PANDA was downregulated in DLBCL patients

**Figure 2:**
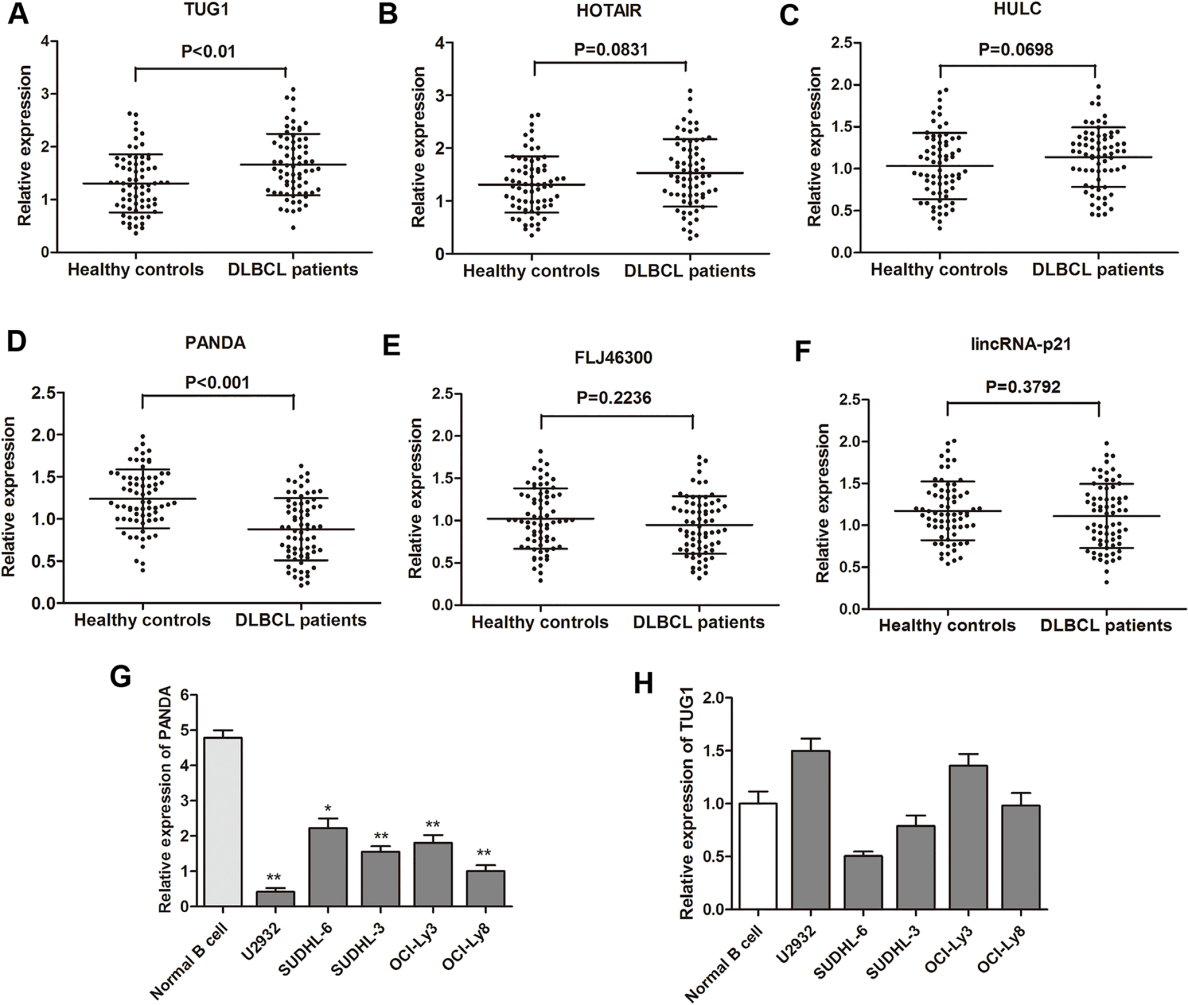
Analysis and validation of selected lncRNA expression by RT-qPCR (**A**–**F**) Concentrations of six identified serum lncRNAs in DLBCL patients (*n* = 68) and healthy controls (*n* = 68) using RT-qPCR assay in validation cohort. (**G**–**H**) The expression levels of lncRNA PANDA and TUG1 were further validated in five DLBCL cell lines and one normal cell line.

RT-qPCR was performed to further test the 15 lncRNAs selected through the Hiseq sequencing method by using 40 primary DLBCL tissues and 40 noncancerous lymph node tissues. Among these, six lncRNAs (TUG1, HOTAIR, HULC, PANDA, FLJ46300, LincRNA-p21) were found significantly dysregulated (Table 2). Subsequently, these six genes were further determined by RT-qPCR in the validation phase containing 68 serum samples from patients with DLBCL and 68 from healthy controls. In this phase, we observed that one lncRNA PANDA was significantly down-regulated in DLBCL patients compared with healthy controls, while another lncRNA TUG1 expression was dramatically increased in DLBCL patients (Figure 2A). Then, we detected the expression of lncRNA PANDA and TUG1 in DLBCL cell lines. We found that PANDA was significantly down-regulated in the five DLBCL cell lines compared with normal cells, while no statistical significance was found for the expression of TUG1 in different cell lines (Figure 2B). More importantly, previous studies showed that PANDA may participated in tumorigenesis of several malignancies through the regulation of cell proliferation [15]. Thus, we focus on the clinical and experimental role of lncRNA PANDA in DLBCL.

**Table 2:** Expression of 15 candidate lncRNAs in tissue specimens from DLBCL patients and control individuals [median (interquartile range)]

LncRNA	DLBCL	Controls	*P* value
TUG1	2.41 (1.30–3.44)	1.21 (0.40–2.85)	< 0.01
FIRRE	1.35 (0.49–3.08)	0.98 (0.30–2.77)	0.10
SBDSP1	1.64 (0.56–3.28)	1.09 (0.36–2.44)	0.25
GGT8P	1.61 (0.72–3.71)	1.32 (0.30–2.05)	0.34
MIR210HG	1.29 (0.93–2.57)	1.01 (0.41–2.05)	0.12
FLJ46300	0.77 (0.45–1.93)	1.36 (0.42–2.06)	< 0.01
PANDA	1.02 (0.39–2.81)	2.56 (0.53–3.88)	< 0.01
FTX	1.32 (0.28–2.33)	1.77 (0.65–3.01)	0.09
LOC151009	0.98 (0.41–1.99)	1.23 (0.89–2.42)	0.17
LOC202181	0.88 (0.30–1.51)	1.30 (0.41–2.55)	0.10
HOTAIR	1.63 (0.43–2.37)	0.83 (0.27–1.74)	< 0.05
HULC	1.62 (0.45–2.18)	0.94 (0.33–1.43)	< 0.05
LUNAR1	0.84 (0.34–2.71)	0.64 (0.23–1.68)	0.29
LINCRNA p21	0.82 (0.22–1.69)	1.56 (0.45–2.83)	< 0.01
PEG10	1.24 (0.55–1.61)	1.02 (0.31–1.78)	0.47

### Decreased expression of serum lncRNA PANDA indicates poor outcome of DLBCL patients

**Figure 3:**
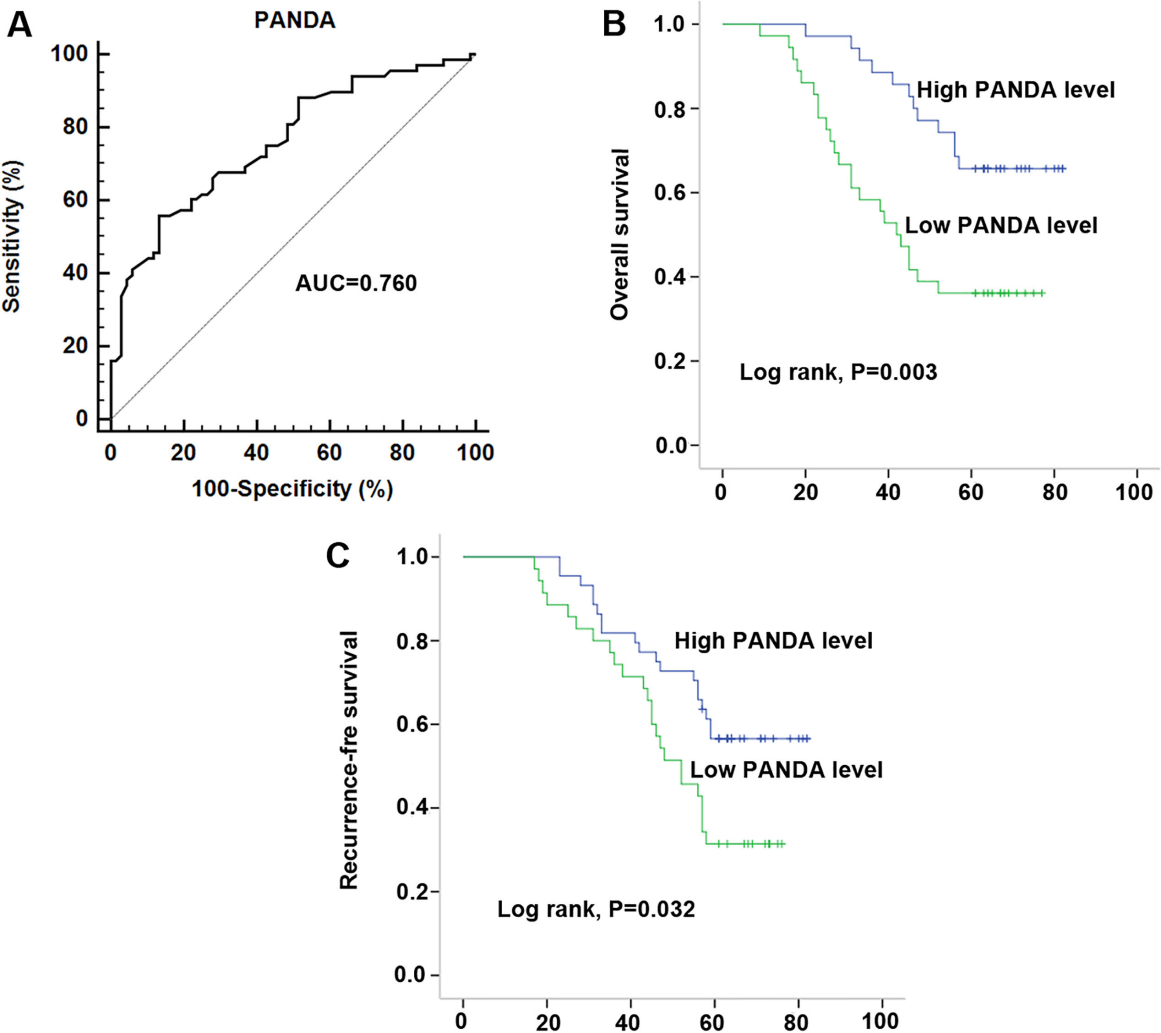
Decreased serum PANDA expression was associated with poor clinical outcome in DLBCL patients (**A**) ROC curves for differentiating the DLBCL patients from healthy controls using lncRNA PANDA expression in validation cohort. (**B**–**C**) Kaplan-Meier curves for overall survival (B) and recurrence-free survival (C) according to serum levels of PANDA in DLBCL patients in validation cohort.

After having validated the down-regulation of lncRNA PANDA, we investigated the association between serum PANDA expression and clinicopathological characters. As shown in Table 3, lncRNA PANDA was significantly correlated with B symptoms, Ann arbor stages, CHOP-like treatment, Rituximab and IPI, while no significant correlations were observed between PANDA expression and other clinicopathological factors, such as gender, age, performance status and subtypes in 68 serum samples. In addition, a similar conclusion was also developed in the 40 primary tissue samples (Table 4). To evaluate the role of PANDA in distinguishing between the DLBCL patients and healthy controls, ROC curve analysis was performed and the cut-off value was established (1.02). The area under the curve (AUC) and diagnostic sensitivity and specificity reached 0.760, 60.29%, and 77.94%, respectively (Figure 3A). Then, Kaplan-Meier survival analysis was performed to investigate the prognostic value of PANDA in DLBCL patients. Our results indicated that patients with high PANDA expression were associated with longer OS and RFS compared with low PANDA patients (Figure 3B and 3C). Furthermore, we performed Cox regression univariate/multivariate analysis to identify whether PANDA or any other clinical parameters were independent indicators for the overall survival of DLBCL patients. The results indicated that PANDA expression level and Ann Arbor stages status were identified as potential independent prognostic factors for overall survival of DLBCL patients (Table 5).

**Table 3:** Association of lncRNA PANDA expression with clinical parameters in DLBCL patients

		total *n*(%)	High PANDA expression *n* (%)	Low PANDA expression *n* (%)	*P* value
**Gender**					0.590
	Male	49	26 (38.2)	23 (33.8)	
	Female	19	8 (11.8)	11 (16.2)	
**Age, year**					0.825
	Median		63	59	
	Range		34–85	36–79	
**B symptoms**					0.024^*^
	Present	26	8 (11.8)	18 (26.5)	
	Absent	42	26 (38.2)	16 (23.5)	
**Ann Arbor stages**					0.014^*^
	I-II	31	21 (30.9)	10 (14.7)	
	III-IV	37	13 (19.1)	24 (35.3)	
**Performance status**					0.803
	0–2	26	14 (20.6)	12 (17.6)	
	3–4	42	20 (29.4)	22 (32.4)	
**CHOP-like treatment**					0.003^*^
	Response	33	23 (33.8)	10 (14.7)	
	Non-response	35	11 (16.2)	24 (35.3)	
**Rituximab**					0.006^*^
	Response	38	25 (36.8)	13 (47.1)	
	Non-response	30	9 (13.2)	21 (19.1)	
**Subtypes**					0.642
	GC	39	18 (26.5)	21 (30.9)	
	Non-GC	29	16 (23.5)	13 (19.1)	
**IPI**					0.021^*^
	0–2	24	7 (10.3)	17 (25.0)	
	3–5	44	27 (39.7)	17 (25.0)	

*, statistical significance.

**Table 4:** Association of lncRNA PANDA expression with clinical parameters in DLBCL tissues

		total *n* (%)	High PANDA expression *n* (%)	Low PANDA expression *n* (%)	*P* value
**Gender**					0.741
	Male	26	12 (30.0)	14 (35.0)	
	Female	14	8 (20.0)	6 (15.0)	
**Age, year**					0.749
	Median		60	61	
	Range		37–82	41–79	
**B symptoms**					0.022^*^
	Present	16	4 (10.0)	12 (30.0)	
	Absent	24	16 (40.0)	8 (20.0)	
**Ann Arbor stages**					0.019^*^
	I-II	14	11 (27.5)	3 (7.5)	
	III-IV	26	9 (22.5)	17 (42.5)	
**Performance status**					0.523
	0–2	17	10 (25.0)	7 (17.5)	
	3–4	23	10 (25.0)	13 (32.5)	
**CHOP-like treatment**					0.001^*^
	Response	21	16 (40.0)	5 (12.5)	
	Non-response	19	4 (10.0)	15 (37.5)	
**Rituximab**					0.001^*^
	Response	23	17 (42.5)	6 (15.0)	
	Non-response	17	3 (7.5)	14 (35.0)	
**Subtypes**					0.515
	GC	25	11 (27.5)	14 (35.0)	
	Non-GC	15	9 (22.5)	6 (15.0)	
**IPI**					0.022^*^
	0–2	14	3 (7.5)	11 (27.5)	
	3–5	26	16 (40.0)	10 (25.0)	

*, Statistical significance.

**Table 5:** Univariate and multivariate Cox proportional hazards regression model analysis of factors for OS in patients with DLBCL

Characteristics	Univariate analysis			Multivariate analysis		
	HR	95% CI	*P* value	HR	95%CI	*P* value
Gender	1.012	0.605–2.035	0.682			
Age	1.511	0.706–2.902	0.157			
Performance status	1.698	0.521–2.906	0.228			
Subtypes	1.883	1.127–3.359	0.069			
IPI	1.577	0.941–2.694	0.139			
Ann Arbor stages	2.780	1.569–3.903	0.009	2.791	1.475–4.462	0.007^*^
B symptoms	2.173	1.133–3.936	0.038	2.202	1.132–3.308	0.058
PANDA expression	1.973	0.368–2.315	0.006	1.893	0.384–2.447	0.010^*^

^*^, Statistical significance.

### LncRNA PANDA is activated by transcription factor p53 and p53 interacts with the promoter region of PANDA

**Figure 4:**
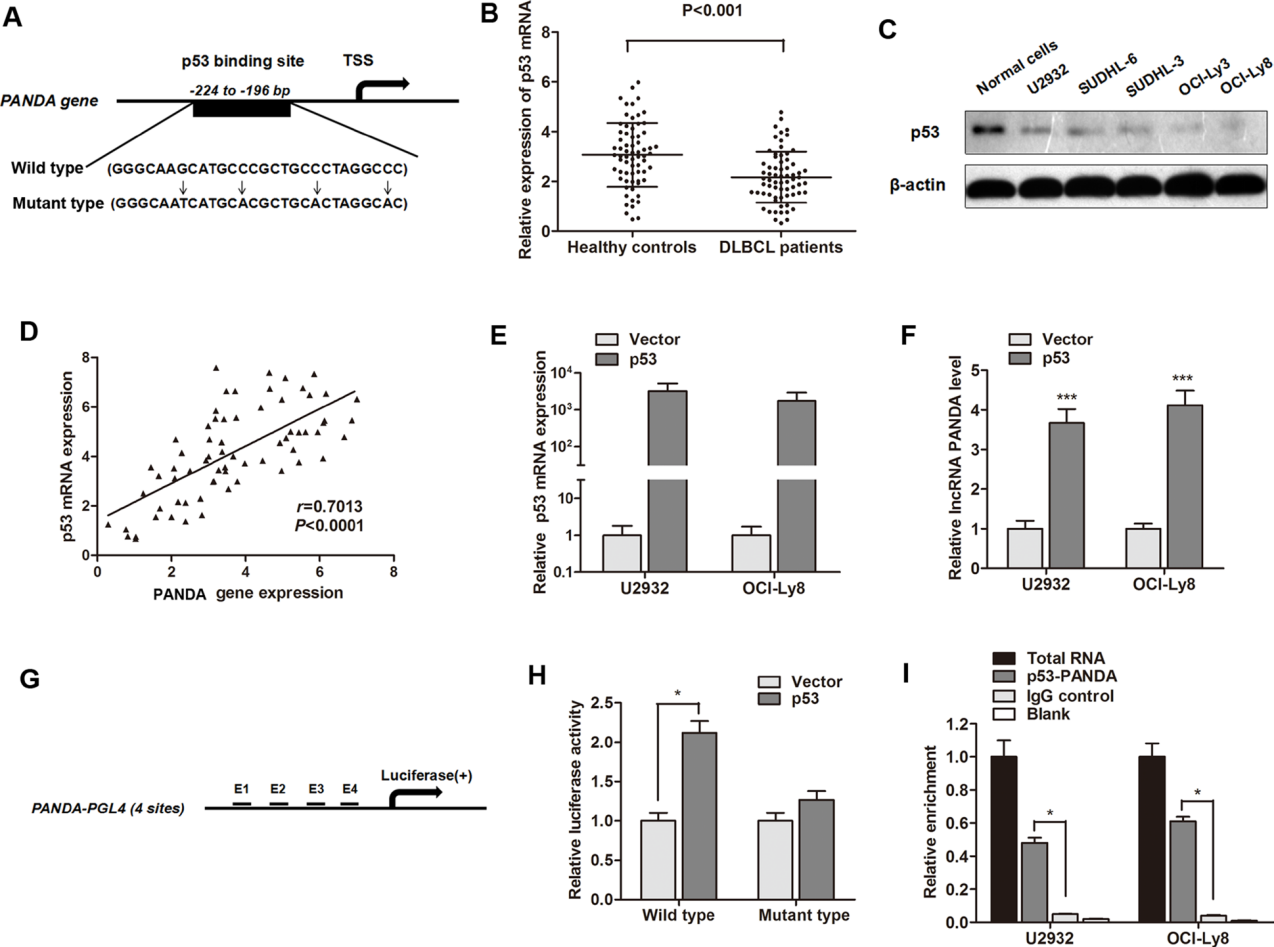
LncRNA PANDA is induced by p53 and p53 interacts with the p53 response element in the promoter region of PANDA (**A**) The potential binding site of p53 at the promoter region of lncRNA PANDA, and the description of wild p53 and mutant p53. (**B**) RT-qPCR showed that p53 mRNA was significantly decreased in serum of DLBCL patients compared with healthy individuals. (**C**) Western blot experiment indicated that p53 protein was dramatically silenced in most DLBCL cell lines compared with normal cells. (**D**) Spearman correlation assay suggested a positive correlation between p53 mRNA and MALAT1expression. (**E**) p53 was significantly elevated by the transfection of p53 overexpression vector. (**F**) lncRNA PANDA was significantly up-regulated by p53. (**G**) Potential p53 binding region in the promoter region of PANDA used for construction of luciferase vector containing the binding region. (**H**) Luciferase activity was significantly increased in p53-transfected cells compared with control vector in OCI-Ly8 cells. (**I**) The p53 binding at the promoter regions of PANDA was assessed by ChIP analysis. Shown are representative images of three independent experiments.

In order to determine why lncRNA PANDA is silenced in DLBCL tissues, we focused on transcription factors that potentially bind to the PANDA promoter. Since p53 is a positive regulator of CDKN1A during the DNA damage response, we asked whether p53 regulates PANDA expression. Based on the computer algorithms PROMO (http://alggen.lsi.upc.es/cgi-bin/promo_v3/promo/promoinit.cgi?dirDB=TF_8.3), and GeneCards (http://www.genecards.org/cgi-bin/carddisp.pl?gene=MYC&keywords=cmyc), we also identified the p53 binding site on the promoter region of PANDA gene (Figure 4A). We then determined the expression of p53 and found that serum p53 mRNA was down-regulated in DLBCL patients compared with healthy individuals (Figure 4B). p53 protein was also down-regulated in DLBCL cell lines compared with normal B-cell lines (Figure 4C). Moreover, RT-qPCR showed a significant positive correlation between p53 and PANDA expression in serum samples of DLBCL patients (Figure 4D). LncRNA PANDA expression was significantly increased after p53 was over-expressed in U2932 and OCI-Ly8 cells (Figure 4E and 4F).

To investigate the direct binding of p53 to the PANDA promoter, we cloned the promoter region (∼1.5 kb) of PANDA into luciferase reporter plasmid (pGL4 basic, Figure 4G). As shown in Figure 4H, luciferase activity was significantly increased in wild p53-transfected cells compared with control vector in OCI-Ly8 cells, however, no change of luciferase activity we found when the cells were transfected mutant-p53 RNA. ChIP experiments showed that a significant increased immunoprecipitation of p53 was identifed at the promoter region of PANDA compared with blank IgG controls (Figure 4I). To conclude, we validated that lncRNA PANDA is activated by transcription factor p53 and p53 can interact with the promoter region of PANDA gene.

### LncRNA PANDA suppresses proliferation and induces cell-cycle arrest in DLBCL cells

**Figure 5:**
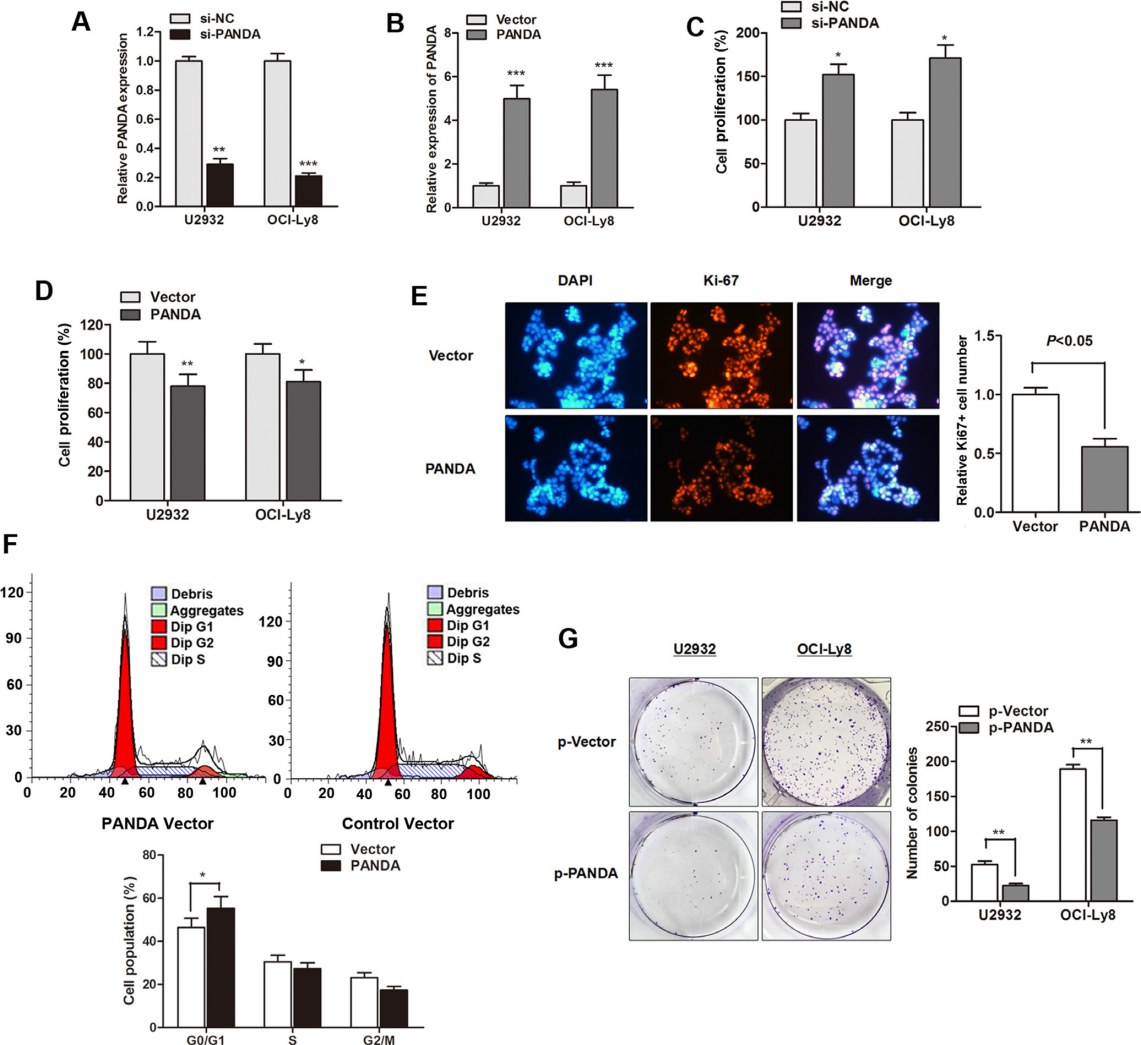
LncRNA PANDA suppresses proliferation and induces cell-cycle arrest in DLBCL cells (**A**–**B**) PANDA expression was significantly silenced by PANDA inhibitor (A) or promoted by PANDA vector (B). (**C**–**D**) si-PANDA significantly promoted cell proliferation rate (C) while PANDA overexpression suppressed cell growth (D). (**E**) Immunofluorescence analysis PANDA overexpression dramatically suppressed the cell proliferation marker Ki-67 protein. (**F**) Cell-cycle analysis indicated that overexpression of PANDA promoted cell cycle arrest in G0/G1 phase in OCI-Ly8 cells. (**G**) LncRNA PANDA suppressed the colony formation capacity of U2932 and OCI-Ly8 cells.

As a follow-up to our patient data that revealed a lower expression of PANDA in DLBCL, we further investigated the biological function of PANDA *in vitro*. LncRNA PANDA was silenced or overexpressed by transfection of si-PANDA or PANDA vector, respectively (Figure 5A and 5B). CCK8 assay showed that si-PANDA significantly promoted cell viability while PANDA overexpression suppressed cell proliferation rate of DLBCL cells (Figure 5C and 5D). We also detected the expression of proliferation marker Ki-67 and found that lncRNA PANDA suppressed Ki-67 expression in OCI-Ly8 cells (Figure 5E). Concomitant with this inhibition of cell proliferation by PANDA, the cell cycle analysis indicated that cell cycle arrest reached significance at the G0/G1 checkpoint after over expression of PANDA (Figure 5F). Moreover, PANDA also suppressed the colony formation capacity of DLBCL cells (Figure 5G). However, no significant effect of PANDA on DLBCL cell apoptosis, migration and invasion was found (Date not shown). This suggests that lncRNA PANDA may act as tumor suppressor gene through regulating cell growth with a G0/G1 cell cycle arrest in DLBCL.

### LncRNA PANDA regulates cell proliferation through inactivation of MAPK/ERK signaling pathway

**Figure 6:**
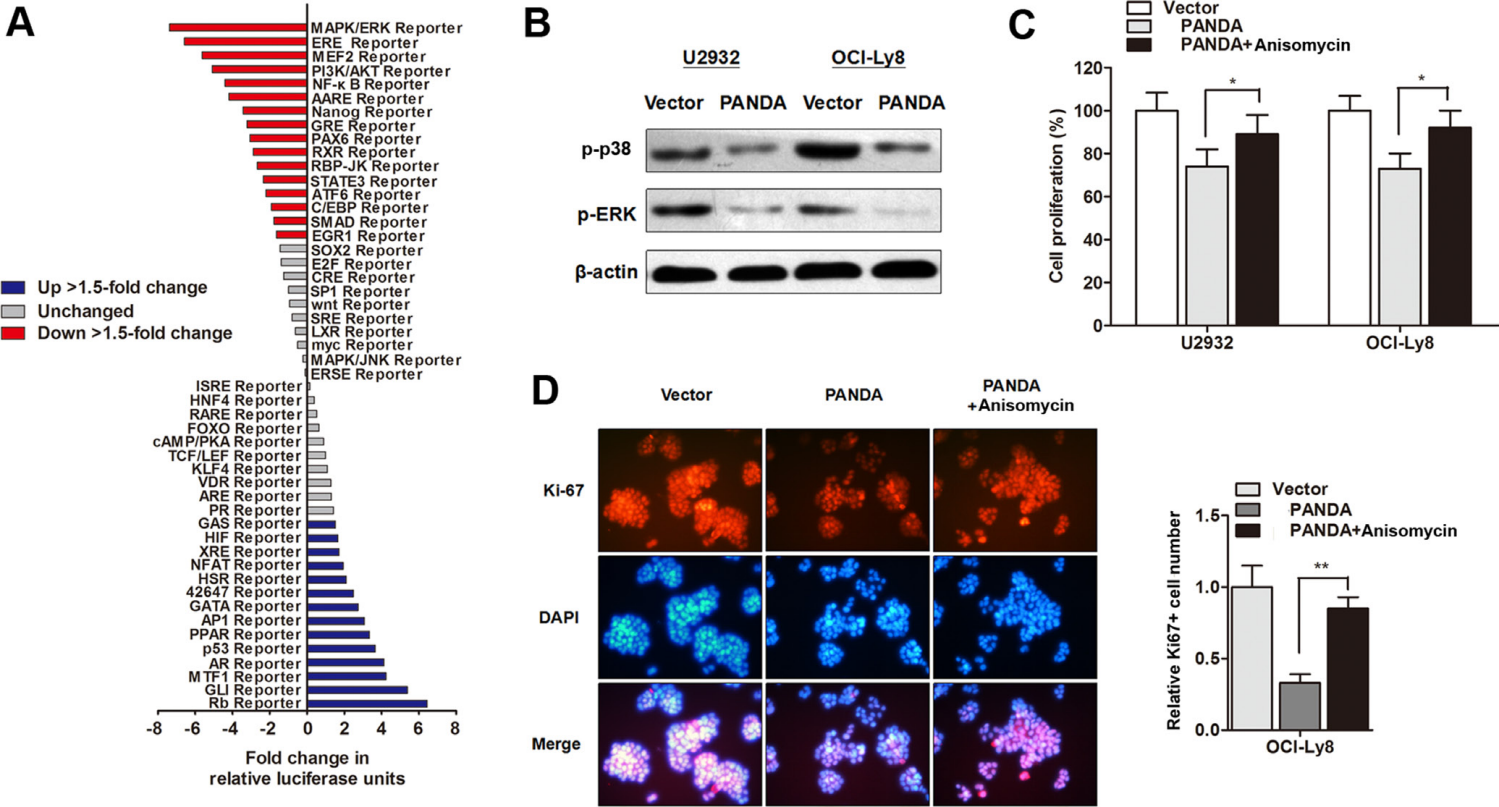
LncRNA PANDA regulates cell proliferation through inactivation of MAPK/ERK signaling pathway (**A**) Histogram shows the fold changes for the activities of different signaling pathways, as indicated by reporter activity. (**B**) Western blot analysis of the expression levels of p38-MAPK and p-ERK protein. (**C**) CCK8 assay showed that treatment with MAPK/ERK agonist Anisomycin potently abolished PANDA-induced suppression of cell growth. (**D**) Cell proliferation marker Ki-67 was suppressed by PANDA, however, this effect was partially reversed by MAPK/ERK agonist Anisomycin.

Subsequently, we investigated the underlying regulatory mechanism by which PANDA regulates DLBCL progression. The Cignal Signal Transduction Reporter Array was performed to determine the change of signaling activity PANDA was overexpressed in OCI-Ly8 cells. Among the 50 candidate signaling pathways, we found that the activity of MAPK/ERK signaling pathway was mostly silenced by PANDA overexpression (Figure 6A). MAPK/ERK signaling pathway participates in the regulation of proliferation and cell cycle in tumors, and it has been well accepted that there are functional interactions between p53 and MAPK/ERK signaling pathway [21]. Herein, we sought to determine if MAPK/ERK pathway is responsible for the lncRNA PANDA-induced suppression of cell proliferation. As expected, western blot experiments showed that lncRNA PANDA inhibited the expression of proteins involved in MAPK/ERK pathway (Figure 6B). In addition, DLBCL cells were treated with MAPK/ERK agonist Anisomycin, and the CCK8 assay showed that treatment with Anisomycin potently abolished the PANDA-induced suppression of cell growth (Figure 6C). Cell proliferation marker Ki-67 was also reversed by Anisomycin, suggesting that lncRNA PANDA may regulate DLBCL cell proliferation via MAPK/ERK pathway (Figure 6D).

## DISCUSSION

LncRNAs has emerged as an important regulator with impact on a wide range of cancers [22]. They have been identified as novel biomarkers and therapeutic targets for various cancers including DLBCL. In current study, high-throughput HiSeq sequencing was firstly employed to search for potential lncRNAs that may help improve the efficacy of diagnosis and prognosis in DLBCL patients. Then, the selected lncRNAs were detected and validated in different specimens such as primary tissues and serums from DLBCL patients, which can assure a relative high accuracy of the date. LncRNA PANDA was then screened out as the most promising lncRNA among the candidates, and its high diagnostic value was also observed in view of high AUC, diagnostic sensitivity and specificity by ROC curves. Additionally, Kaplan-Meier analysis suggested that lower PANDA expression was correlate with poorer survival rate for patients with DLBCL. Moreover, we demonstrated that lncRNA PANDA was induced by p53 and PANDA can suppress cell proliferation through inactivation of MAPK/ERK signaling pathway.

The aberrant expressions of specific lncRNAs in cancer can mark the spectrum of disease progression and may serve as independent biomarkers for diagnosis and prognosis [23]. Previously, Zhou et al. identified a group of lncRNAs that that may have potential value in diagnosting and predicting DLBCL [24]. However, whether some other lncRNAs can also serve as mature biomarkers in DLBCL is still not well known. For this reason, it is of utmost importance to identify molecular bio-markers that have effective diagnostic and prognostic meaning. In this study, we sought to identify one or more lncRNAs that may have function in the formation of DLCBL and its progression by unabridged research strategies. As we know, one lncRNA may expressed different levels in different samples and different diseases. Thus, different clinical materials were used to ensure that the selected lncRNAs are qualified to the use in clinical practice. Moreover, we enrolled a relatively large sample group to ensure the steady expression of candidate lncRNAs. With this unabridged research strategy, we identified only one significantly altered lncRNA, PANDA.

LncRNA PANDA was firstly identified by Hung et al. and located approximately 5 kilobases upstream of the p21 TSS, coincides with a cluster of previously annotated ESTs, and is evolutionarily conserved and potentially regulated by p53 [22]. Specifically, PANDA is a 5′-capped and polyadenylated non-spliced lncRNA that is transcribed antisense to p21 but not dependent on p21. It is reported to repress apoptosis by inhibiting the function of nuclear transcription factor Y subunit alpha (NF-YA). In addtion, it can interact with p53 and stabilize p53 protein in response to DNA damage. p53 is a transcription factor and then induced by cellular stress regulates proliferation, cell cycle arrest and apoptosis [25]. Published data also demonstrated that p53 participates in the regulation of neuronal differentiation. Our date suggests that PANDA is induced by translational factor p53 in DLBCL, and p53 can specifically activate PANDA expression through binding to PANDA promoter region.

Previous studies about the function of PANDA in cancer progression are very limited. Puvvula PK et al. indicated that PANDA can positively regulate cell senescence entry and exit via scaffold-attachment-factor SAFA, however, this does not develop a consistency [26]. To the best of our knowledge, we have reported for the first time that lncRNA PANDA can suppress proliferation of DLBCL cells with a G0/G1 cell cycle arrest manner, but had little effect on apoptosis and metastasis. To reveal how lncRNA PANDA participates in the regulation of cell proliferation, we performed Cignal Signal Transduction Reporter Array. This array involved a mixture of a pathway-specific transcription factor-responsive firefly luciferase reporter, which contains a specific transcription factor-responsive element in the promoter. This high-throughput dual-luciferase assay leads us to identify MAPK/ERK pathway as one putatively affected by lncRNA PANDA. There are three major subfamilies of MAPKs, including p38, ERKs, JNKs, which positively participated biological processes such as migration, proliferation and angiogenesis. The MAPK/ERK signaling pathway is a main signal transduction pathway closely connected with several stress reactions, and physical and chemical reactions within the cells. It was involved in the cell response to outside stimuli by activating and regulating the client protein [27]. Moreover, a wide range of studies have found that the MAPK/ERK pathway closely interacted with p53 pathway [28], which is consistent with the date obtained from the high-throughput dual-luciferase assay.

Aberrant activation of the MAPK/ERK signaling pathway has been observed in many types of human cancers including DLBCL [29]. It mediates cancer cell invasion and proliferation by orchestrating several key biological processes during the development and progression of cancer [30]. For example, inhibition of MAPK signaling in breast cancer impairs proliferation and promotes apoptosis [31]. Interestingly, this signaling pathway are reported to be regulated by another lncRNA, MALAT1. Han et al. demonstrated that lncRNA MALAT1 can suppress glioma progression through down-regulation of MMP2 and inactivation of MAPK/ERK pathway [32]; Chen et al. revealed that lncRNA MALAT1 promoted the activation of MAPK/ERK signaling pathway in N2a cells [33]. These suggest that the MAPK/ERK pathway may also be regulated by other lncRNAs. In our study, we demonstrates that lncRNA PANDA inhibits DLBCL cell growth through the inactivation of MAPK/ERK signaling pathway.

The results of the present study were a little different from others. LncRNA PANDA was reported to be a potential tumor promoter gene in osteosarcoma [15]. But in the present study, it was proved that PANDA acts as a tumor suppressor gene. It may be caused by limited samples or the different types of tumors. Hence, more researches of this lncRNA in other cancers are needed. In conclusion, this is, to our knowledge, the first description of the role of lncRNA PANDA in DLBCL progression. The integrated approach reveals that PANDA is down-regulated in DLBCL patients compared with healthy individuals, and closely associated with clinical prognosis. Moreover, it suppresses cell proliferation and caused cell-cycle arrest through inactivation of MAPK/ERK signaling pathway. Hence, PANDA may be a promising therapeutic target for patients with DLBCL.

## MATERIALS AND METHODS

### Study design

A multiphase, case–control study was designed to identify lncRNAs as potential biomarkers for differentiating DLBCL and healthy people. Briefly, 114 patients diagnosed with DLBCL but without other diseases and 114 control individuals without the history of DLBCL were recruited from The First Affiliated Hospital of Zhengzhou University. All these participants were allocated to three phases. In the initial screening phase, six serum samples from DLBCL patients and six from healthy controls were subjected to Hiseq sequencing, to identify lncRNAs that significantly differentially expressed. In the training phase, the candidate lncRNAs were firstly verified with RT-qPCR in an independent cohort of 40 primary DLBCL tissues from DLBCL patients and 40 normal lymph node tissues from control individuals with reactive lymph. In the validation phase, the candidate lncRNAs were further validated in another independent group of serum samples obtained from 68 DLBCL patients and 68 controls individuals. The control samples enrolled in this study are matched to the patient samples (tissues and serums) on clinical pathological charicteristics, such as gender and age.

### Patients and sample preparation

All the patients were pathologically confirmed and they were classified according to the tumor-node-metastasis (TNM) classification. Overall survival (OS) was updated on 1 February 2012 and was defined as the time from inclusion to death for any reason. Recurrence free survival (RFS) was defined as the time from inclusion to recurrence or metastasis progression. After ultrasonic biopsy, tissues specimens were immediately frozen at −80°C until RNA extraction. Venous blood was collected and centrifuged at 4000 rpm for 10 min, within 2 h. The supernatant fluids were then collected and further centrifuged at 12000 rpm for 15 min to completely remove the cell debris. The whole process was strictly controlled to avoid hemolysis, and the supernatant serum was stored at −80°C, until further used.

### Hiseq sequencing

Total tissue RNA was extracted by one-step extraction using a Trizol kit (Life Technologies, USA), and the purity and quantity of RNA were determined by UV spectrophotometry. cDNA library construction and sequencing were performed according to previously described methods (9). Briefly, after extraction of total RNA, ribosomal RNA was separated to isolate as ncRNA as possible. RNA fragments were broken into short fragments randomly. The first chain of cDNA was generated using RNA fragments as templates and 6-bp random primers. Second chain of the cDNA was synthesized according to the kit's instruction (TakaRa Co., Ltd., Dalian, China). After purification, end repair, base A and sequencing joint adding, the generated cDNA was fragmented using uracil-N-glycosylase (UNG). cDNA fragments were chosen according to size, then PCR amplification was performed to establish the complete sequencing cDNA library. lncRNAs were sequenced using the high-throughput, high-sensitivity HiSeq 2500 sequencing platform (Illumina Company, USA). The whole process and subsequent data analysis were performed by Kangchen Bio-tech, Shanghai, P.R. China. FastQC software was used for quality control of the pretreated data.

### Cell culture

The human DLBCL cell lines (U2932, SUDHL-6, SUDHL-3, OCI-Ly3, and OCI-Ly8) and normal B-cell line (WIL2S) were obtained from Type Culture Collection of the Chinese Academy of Sciences (Shanghai, China). DLBCL cells were cultured in RPMI 1640 (Thermo Fisher Scientific, Wilmington, DE, USA) containing 10% fetal bovine serum (FBS) (Sigma-Aldrich, St. Louis, MO, USA), 100 U/ml penicillin, and 100 g/ml streptomycin (Life Technologies, Grand Island, NY, USA). B-cell lines were maintained in Iscove's modified medium supplemented with 10% human serum (NABI Biopharmaceuticals, Boca Raton, FL, USA), 100 U/ml penicillin, 100 mg/ml streptomycin, and 2 mM l-glutamine (Invitrogen, Carlsbad, CA, USA). All the cells were incubated in a humidified atmosphere at 37°C in 5% CO_2_ and 95% air. The MAKP/ERK agonist Anisomycin was bought from Sigma-Aldrich (St. Louis, MO, USA) and used as instructions.

### RNA oligoribonucleotides and cell transfection

The small interfering RNAs (siRNA) that specifically target human lncRNA PANDA were designated as si-PANDA (Genechem corporation, Shanghai, China). The lncRNA PANDA overexpression plasmid (PANDA vector), p53 overexpression plasmid or control vector was purchased from Addgene. DLBCL cells were plated in 24-well plates at 1 × 10^5^ per well. Forty-eight hours after plating, 100 nM of RNA oligoribonucleotides were transfected into the cells with Lipofectamine 2000 (Invitrogen) according to the manufacturer's instructions.

### RNA extraction, reverse transcription, and RT-qPCR

Total RNA was isolated from primary DLBCL tissues or cell lines using TRIzol reagent (Invitrogen). And then, the cDNA was synthesized from 200 ng extracted total RNA using the PrimeScript RT reagent Kit (Takara Bio Company, Shiga, Japan) and amplified by RT-qPCR with an SYBR Green Kit (Takara Bio Company) on an ABI PRISM 7500 Sequence Detection System (Applied Biosystems, Foster City, CA, USA) with the housekeeping gene GAPDH as an internal control. The 2^−ΔΔCt^ method was used to determine the relative quantification of gene expression levels. All the premier sequences were synthesized by RiboBio, and the premier sequences were as follows: PANDA: (Forward) 5′-AGACCCCAGTGGCACCTGAC-3′, (Reverse) 5′-GG GCAGAACTTGGCATGATG-3′; p53 (Forward) 5′-GTCG ATCGTCGATCGCTACGC-3′, (Reverse) 5′-CGTAG CTAGTCGATCGACTAGC-3′; GAPDH (Forward) 5′-G CACCGTCAAGGCTGAGAAC-3′, (Reverse) 5′-ATGGT GGTGAAGACGCCAGT-3′. Each experiment was performed in triplicate.

### Signal transduction reporter array

Cignal Signal Transduction Reporter Array (Qiagen, Valencia, CA, USA) was used to simultaneously investigate alternations in the activities of 50 canonical signalling pathways in response to UCA1 knockdown. Cells were transfected with antisense oligonucleotides-targeting UCA1 for 24 h and were subsequently transfected with a mixture of a transcription factor-responsive firefly luciferase reporter and a constitutively expressing Renilla construct. The relative activity of each pathway was decided by luciferase/Renilla and normalized by untreated controls. Experiments were performed in triplicates.

### Cell proliferation assay

Cell proliferation was quantified by using the Cell Counting Kit-8 (CCK-8, Beyotime Corporation, Shanghai, China). Briefly, 100 μl of cells from the different transfection groups were seeded onto a 96-well plate at a concentration of 2000 cells per well and were incubated at 37°C. At different time point, the optical density was measured at 450 nm using a microtiter plate reader, and the rate of cell survival was expressed as the absorbance. The results represent the mean of three replicates under the same conditions.

### Cell cycle assay

Cells were washed in PBS and fixed in 70% ethanol at 4°C for 2 h. DNA staining was done with 10 mg propidium iodide/mL PBS and 2.5 Ag DNase-free RNase/mL PBS for at least 30 min before flow cytometry in a Coulter EPICS XL flow cytometer (Beckman Coulter, Inc., Fullerton, CA). Cell cycle profiles were generated from flow cytometry analysis with Modifit software (BD Biosciences).

### Immunofluorescence analysis

DLBCL cells were grown to 40% to 50% confluence and then transfected with 100 nM of si-PANDA or PANDA overexpression vector. After 48 hours of incubation, the cells were fixed with 4% paraformaldehyde and permeabilized in 0.2% Triton X-100 (Sigma-Aldrich) for 20 minutes. The cells were then blocked with 10% goat serum in PBS for 1 h. Cells were incubated with primary anti-Ki-67 (Cell Signaling Technology) overnight at 4°C and then incubated with the appropriate rhodamine-conjugated secondary antibody for 1 h. The cells were then washed and incubated with DAPI (Invitrogen) for nuclear staining. The slides were visualized for immunofluorescence with a laser scanning Olympus microscope.

### Western blot and antibodies

The primary antibodies were rabbit anti-human p53 antibody (#9282T, 1:1000; Cell Signaling Technology, Beverly, MA, USA), p38-MAPK antibody (#8690T, 1:1000; Cell Signaling Technology), rabbit anti-human phospho-ERK1/2 antibody (#4695T, 1:1000; Cell Signaling Technology), and rabbit anti-human β-actin antibody (#4970T, 1:1000; Cell Signaling Technology). Horseradish peroxidase-conjugated (HRP) anti-rabbit antibodies (1:5000; Santa Cruz Biotechnology, Santa Cruz, CA, USA) were used as the secondary antibodies. Cell lysates in 1× SDS loading buffer (60 mM Tris–HCl, pH 6.8; 2% SDS; 20 % glycerol; 0.25 % bromophenol blue; and 1.25 % 2-mercaptoethanol) were incubated at 100°C for 10 min to facilitate sample loading for conventional western blotting analysis. The relative protein levels were quantified using densitometry with a Gel-Pro Analyzer (Media Cybernetics, Rockville, MD, USA).

### Chromatin immunoprecipitation (ChIP)

ChIP was performed using the EZ ChIP^TM^ Chromatin Immunoprecipitation Kit (Millipore, Bedford, MA, USA), according to the manufacturer's protocol. Briefly, cross-linked chromatin was sonicated into 200–1000 bp fragments. The chromatin located on the promoter of lncRNA PANDA was immunoprecipitated by using anti-p53 (Cell Signaling Technology) antibodies. An isotype-matched IgG was used as a negative control, and the total RNA that immunoprecipitated by p53 antibody served as a positive control. RT-qPCR was conducted to detect the relative enrichment of the lncRNA PANDA promoter. The ChIP primer sequences are as follows: (forward) 5′-GCCCACATAAACACCTCACAAATGA-3′ and (reverse) 5′-CCT TGGAAGCCGGGAGTAGCT-3′ were used for amplify the promoter region of PANDA.

### Statistical analysis

The differences of lncRNA or mRNA expression level between different groups were analyzed by the Mann-Whitney *U*-test or Kruskal-Wallis test. A log-rank test was used to analyze the statistical differences in survival as deduced from Kaplan-Meier curves. Count dates were described as frequency and examined using Fisher's exact test. All differences were regarded as statistically significant when *P* < 0.05. Statistical analyses were performed with GraphPad Prism 5.01 (GraphPad Software, La Jolla, CA, USA).
